# Treatment of Obstructive Sleep Apnea Using Oral Appliances in Saudi Arabia: Are We Following the Guidelines?

**DOI:** 10.3390/healthcare10112220

**Published:** 2022-11-06

**Authors:** Suliman Alsaeed, Farraj Albalawi, Abdulrahman A. Alghulikah, Ghadah Aldakheel, Bassam Alalola

**Affiliations:** 1 Preventive Dental Sciences, College of Dentistry, King Saud bin Abdulaziz University for Health Sciences, Riyadh 14611, Saudi Arabia; 2 King Abdullah International Medical Research Center, Riyadh 14611, Saudi Arabia; 3 Ministry of the National Guard—Health Affairs, Riyadh 14611, Saudi Arabia; 4 General Department of Medical Services, Ministry of Interior, Riyadh 13321, Saudi Arabia

**Keywords:** dental sleep medicine, obstructive sleep apnea, oral appliances, knowledge, attitude

## Abstract

Background: This study aimed to assess sleep medicine physicians’ knowledge and attitudes toward the role of oral appliances (OAs) in managing obstructive sleep apnea (OSA) in Saudi Arabia. Methods: An online questionnaire was administered to the registered physicians practicing sleep medicine (otolaryngology, internal medicine, pulmonology, and family medicine) in Saudi Arabia. The questionnaire included 26 questions under five domains. All the collected data were analyzed using descriptive statistics and Chi-square tests. Results: One hundred sleep physicians (43% Saudi, 75% male) from Saudi Arabia participated in this study. Almost 43% of participants reported inadequate knowledge of the treatment of OSA patients using OAs. Half of the participants were unaware of how OAs work in managing OSA. Most physicians (62%) never referred the patient for treatment of OSA using OAs, whereas 4% referred at least one patient every month. The majority (60%) believed that there are barriers to referring a case for OA treatment, mainly the lack of knowledge of the suitable cases (60%) and the lack of qualified dentists in this area (43%). Conclusion: Most sleep physicians reported poor knowledge of OA indications and mechanism of use, with most never referring a case for OA therapy.

## 1. Introduction

Obstructive sleep apnea (OSA) is the most common sleep-related breathing disorder [1]. It is characterized by the upper airway’s recurrent partial or complete collapse, oxygen desaturation, and sleep fragmentation. OSA results in significant cardiovascular and metabolic consequences and substantially impacts society. Individuals with OSA often present with reduced quality of life, excessive daytime sleepiness, and an increased risk of motor vehicle accidents [2,3,4]. It is estimated that OSA affects 900 million middle-aged adults globally [5]. In Saudi Arabia, it is estimated that 25% of the population between the ages of 30–69 years old suffer from OSA [5]. 

The first line of treatment for OSA is continuous positive airway pressure (CPAP). However, patient acceptance and adherence are often poor, resulting in low effectiveness of treatment [2,6]. Oral appliances fabricated by qualified dentists in dental sleep medicine (DSM) for patients unable to tolerate CPAP or prefer an alternative therapy are recommended in such cases [4,7]. 

DSM is a well-established field in various parts of the world. The American Academy of Dental Sleep Medicine (AADSM) was established in 1991 to promote dentists’ knowledge in sleep medicine. A few years later, the American Academy of Sleep Medicine (AASM) published its first position statement, indicating the role of dentists in the management of OSA. Since then, the number of certified dentists and the clinical use of OAs for the management of snoring and OSA have grown exponentially [7]. 

Dentists have an essential role in identifying individuals with OSA by recognizing anatomic risk factors or using screening questionnaires [8]. In sleep medicine, screening and treatment using OAs are the two main areas dentists can be involved in [7]. When diagnosing OSA, the patients must have a face-to-face evaluation by a sleep physician for a more comprehensive assessment [7,9]. Treatment using OAs cannot be performed without prior consultation and approval of the patient’s sleep physician. Currently, there are no data in Saudi Arabia concerning the knowledge and perception of physicians toward the use of OAs in managing OSA or primary snoring. Hence, this study aimed to assess sleep medicine physicians’ knowledge and attitudes toward the role of oral appliances (OAs) in managing obstructive sleep apnea (OSA) in Saudi Arabia.

## 2. Materials and Methods

### 2.1. Study Design

It was a cross-sectional study conducted among physicians in the sleep medicine field in Saudi Arabia. This study was approved by the Institutional Review Board of King Abdullah International Medical Research Center Riyadh, Saudi Arabia (reference number SP20-321-R). 

### 2.2. Study Instrument

After reviewing the literature, the investigators prepared a structured, self-administered, and close-ended questionnaire. The validity of the questionnaire was established by taking expert opinion from the orthodontist specializing in sleep medicine. The questionnaire pilot was tested on ten physicians to assess its reliability. After receiving their responses, the questionnaire was modified and finalized. An electronic questionnaire was formulated using Google Forms (Google Inc., Mountain View, CA, USA). 

The final questionnaire was emailed to the sleep medicine physicians registered in the Saudi commission for health specialties. The specialties concerned with sleep medicine include otolaryngology, internal medicine, pulmonology, and family medicine. 

The questionnaire consisted of 26 closed-ended questions in the following categories: (1) demographic data, (2) specialty, experience, and clinical rank, (3) knowledge of dental sleep medicine, (4) perception of the effect of OAs on OSA, and (5) referral protocols for OAs to treat OSA. The study was conducted from September 2020 to January 2021.

### 2.3. Data Analysis

Descriptive statistics were reported as means and standard deviations. The Chi-square test was used to analyze the significance of differences among groups. The z test with Bonferroni correction was used to compare column proportions. All statistical analyses were performed using SPSS version 25 data processing software (IBM Corp. Armonk, NY, USA), and the significance level for all tests was set at *p* < 0.05.

## 3. Results

**Figure 1:**
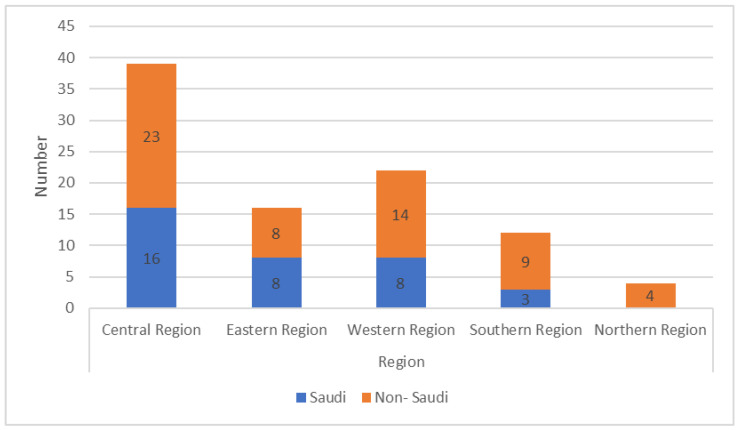
Distribution of respondents from different regions of Saudi Arabia.

**Figure 2:**
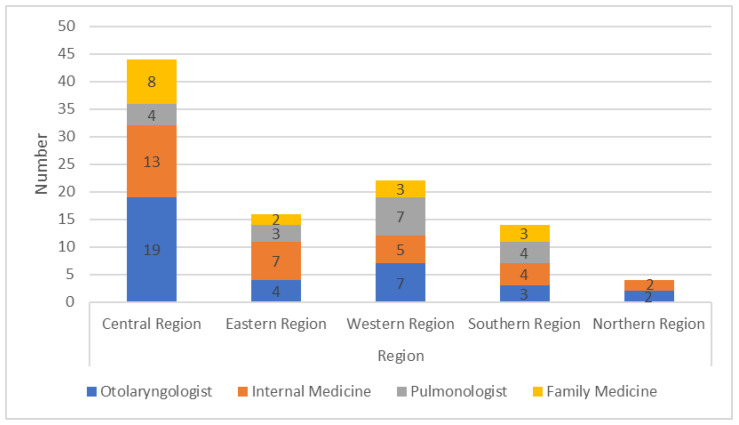
Distribution of specialties across the different regions.

One hundred sleep physicians (43% Saudis, 75% males) from all five regions of Saudi Arabia participated in this study. Most respondents were from the central region (44%), while only 4% were from the northern region (Table 1) (Figure 1). The respondents included physicians specialized in otolaryngology (*n* = 35), internal medicine (*n* = 31), pulmonology (*n* = 18), and family medicine (*n* = 16) and were distributed among the five regions (Figure 2). Most of the participants were specialists (*n* = 37), followed by consultants (*n* = 35) and residents (*n* = 28). Although all participating physicians treat OSA in their practice, only 9% had a fellowship in sleep medicine. The majority (64%) had come across the topic of treatment of sleep apnea using oral appliances (Table 1). 

Regarding their knowledge of the treatment of OSA using OAs, 43% of the participants ranked their knowledge as inadequate (Table 2). Almost half of the participants were unaware of how oral appliances work in managing OSA. In addition, the majority (87%) did not know any qualified dentist in the field of dental sleep medicine (Table 2). However, respondents who had come across the topic of OAs (64%) were more likely to know a dentist who was qualified to manage OSA (*p* = 0.025).

Most physicians (86%) believed in the role of dentists in OSA cases (Table 2). However, 14% of participants, especially those who had an experience of fewer than 5 years in their field (*p* = 0.003), did not believe dentists had a role in helping patients with OSA.

The majority believed in the role of dentists in the field of sleep medicine by screening for OSA patients (52%), treating OSA using oral appliances (70%), or providing lifestyle advice to OSA patients (25%). Only 2% considered dentists having a role in managing OSA by jaw surgery or growth modification of maxillary constrictions. 

**Figure 3:**
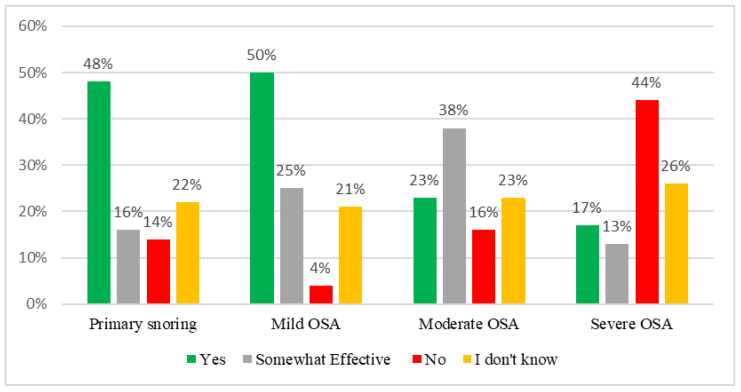
Physicians’ responses to their perception of the effectiveness of OAs.

Regarding the effectiveness of OAs, nearly half (48–50%) of physicians believed that OAs could be effective in treating primary snoring or mild OSA cases. However, 23% and 17% thought OAs were effective in moderate or severe OSA (Figure 3).

The rate of reported referral for OAs was meager, as most physicians (62%) have never referred for OAs, while only 4% have referred at least one patient every month. When evaluating factors influencing patients’ referral frequency for OAs, it was observed that awareness of the topic, reported knowledge of OAs, and knowing a dentist qualified in OSA were significant positive predictors of the frequency of referral of patients. The regression model also showed that perceptions of side effects or knowledge of the mechanism of action of OAs had no impact on the frequency of referral (Table 3).

**Figure 4:**
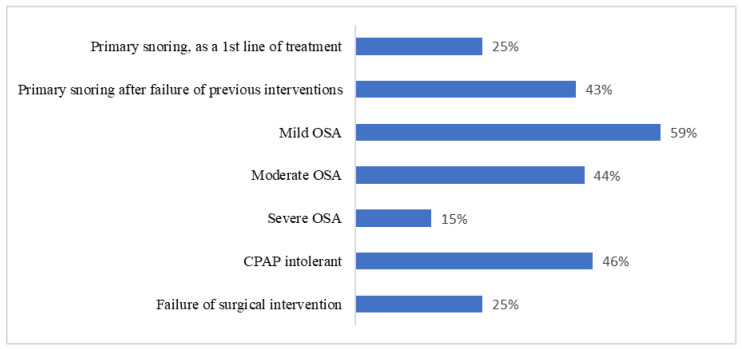
Participants’ responses to which cases they consider for referral for OAs (*n* = 38).

The reported criteria that physicians consider before referral for OAs were reported to be physical examination (67%), sleep study results (65%), and failure of treatment or CPAP intolerance (45%). While 70% of physicians believed OAs could help reduce the required CPAP pressure, only 34% of physicians considered oral appliances for patients treating OSA with high CPAP pressure. Of those who referred at least one case for OAs, their most-referred cases were mild OSA (59%) or CPAP intolerance (46%) (Figure 4).

In response to the question “Which of the following is your usual practice for patients whom you consider suitable for oral appliances?”, 31% of the physicians answered, “No involvement to be performed by the physician”, while 28% decided to refer the case to an oral and maxillofacial surgery clinic (OMFS), and only 21% would refer the patient to an orthodontist or dentist. 

Most physicians (60%) believed there were barriers in referring a case for OA treatment. The most common two barriers were the lack of knowledge on the cases suitable for oral appliances (60%) and the lack of qualified dentists in this area (43%). Only 40% of the physicians were aware of the side effects of oral appliances.

## 4. Discussion

This study aimed to explore the knowledge and attitude of physicians toward the use of oral appliances for the treatment of OSA. A total of 100 physicians from the five regions of Saudi Arabia participated in this study. Although all participating physicians have previously treated OSA patients, almost half of them ranked their knowledge of OAs for OSA patients as inadequate. Regarding their attitude toward OAs, the majority (63%) have never referred a single case for OAs. The rest of the participants rarely refer a case to dentists, as data show that only 4% have referred at least one patient every month. These findings reflect a significant flaw in the management protocol of OSA patients in Saudi Arabia. Hence, this study aimed to shed light on this overlooked treatment option for OSA and identify the different barriers between concerned health practitioners in treating OSA patients. 

During the past 20 years, interest in OSA disorders has increased significantly, with remarkable advancement in clinical and scientific areas [10]. This disorder has been overlooked and underrated due to the lack of knowledge and related health consequences [11]. It has been reported that academic curriculums in Saudi Arabia (SA) medical schools were significantly weak in the area of OSA [12]. With this shortage in our academic curriculums, it is not surprising that the public knowledge of OSA was generally weak, but it is even worse in SA compared to other countries [13]. This poor global awareness of OSA could be one of the reasons why almost 80% of those with moderate to severe OSA in countries such as the United States of America are left undiagnosed [14,15,16]. In SA, a recent study estimated that 25% of the Saudi population between the ages of 30 and 69 suffer from OSA [5]. These alarming facts about OSA led the Saudi Commission for Health Specialties to announce a new Saudi Board Program of Sleep Medicine starting in 2022, aiming to raise standards of health care in sleep medicine, in which OAs can be provided as a treatment option [17]. 

The history of this treatment modality (OAs) goes back to 1902, and it was first recommended by the AASM guidelines in 1995 for the treatment of OSA [18,19], and further updated recommendations were published in 2015 [7]. There are two types of OA: mandibular advancement devices (MADs) and tongue-retaining devices (TRDs) [20]. MADs work by keeping the lower jaw in a forward position, which mechanically allows for a larger airway space, while TRDs work by keeping the tongue in a forward position to open the airway. MADs are more common and effective than TRDs and have more compliance rates than TRDs [20]. There are few studies in the literature on TRDs compared to MADs [20]. The 2015 guidelines recommend that sleep physicians prescribe OAs in cases where patients are intolerant to CPAP instead of no treatment [7]. It is also recommended to refer the case to a qualified dentist in the field of DSM [7]. The guidelines also state that dentists must use custom and titratable devices instead of ready-made or untitratable devices. It is essential to ensure the high effectiveness of the treatment [14].

OAs have been proven to be as effective as CPAP in reducing high blood pressure for OSA patients [21]. One of the main advantages of OAs over CPAP is the higher compliance rate of OAs compared to CPAP [22]. Studies have found that only 43% of OSA patients use CPAP for more than 4 h after six months of treatment, while a 76% compliance rate was recorded for patients wearing OAs [22]. Although it is known that CPAP is more efficient than OAs, the difference in compliance rate led to similar effectiveness between CPAP and OAs [23]. Hence, a team approach must be used whenever a protocol is set for OSA patients to ensure the high effectiveness of treatment. 

Even though the original guidelines go back to 1995, the medical care of OSA patients in SA suffers from a significant flaw in the treatment protocol OSA because most sleep physicians have never referred a single case to a dentist for OAs. This is not surprising, given that around 40% of the participating physicians have never encountered the topic of OA treatment for OSA patients. One of the significant findings of this study is that 14% of the participants did not believe that dentists have any role whatsoever in helping OSA patients. This reflects the severe lack of knowledge of OA for some of the participating physicians. Although some physicians were aware of the suitability of cases with primary snoring, mild OSA, or CPAP intolerance cases for OAs, their knowledge was not reflective of their attitude, as most do not refer for OAs, although they acknowledged it was indicated. Moreover, when the participating physicians were asked about their referral route of cases suitable for OAs, the majority referred to OMFS and only 21% of the time to an orthodontist or a dentist, which is the appropriate referral route when OAs are indicated. In contrast, referral to OMFS is indicated in cases suitable for maxillomandibular advancement (MMA) [7]. 

When the barriers to referrals to dentists were discussed, it was noted that many physicians pointed out that the lack of qualified dentists in SA in this area of sleep medicine is one of the significant barriers. It was also mentioned that most were unaware of cases indicated for OAs. Therefore, the concerned academician in the field of sleep medicine should consider these barriers to educate the involved health care providers on the role of dentists and the use of OAs for OSA patients. 

Although 100 physicians participated in the study, reaching this number of responses was challenging given the shortage of sleep physicians in Saudi Arabia, especially in the northern and southern regions of SA. In addition, this study is at risk of selection bias due to the different sampling techniques in which many institutions were contacted for sleep physicians to participate. 

## 5. Conclusions

- The study participants demonstrated poor knowledge of oral appliance’s indication and mechanism of use.
- Almost half of the physicians consider their knowledge of OAs inadequate.
- Significant flaws were identified in the treatment protocol for cases indicated for OAs.
- Most physicians have never referred a single case to dentists for OAs.
- The physicians listed many barriers to referrals to dentists, including their lack of knowledge of OAs and the lack of qualified dentists in Saudi Arabia.
- The healthcare system related to sleep medicine should educate physicians on the role of dentists in OSA and the appropriate referral routes to aid in providing patients with high standards of health care.

## Figures and Tables

**Table 1:** Demographic and experience level of participants (*n* = 100).

Variable		*n*
Nationality	Saudi	43
	Non-Saudi	57
Gender	Male	75
	Female	25
In which region of Saudi Arabia do you practice?	Central Region	44
	Western Region	22
	Eastern Region	16
	Southern Region	14
	Northern Region	4
Please indicate your specialty	Otolaryngologist	35
	Internal medicine	31
	Pulmonologist	18
	Family Medicine	16
What is your clinical rank?	Consultant	35
	Specialist	37
	Resident	28
Do you have a fellowship in sleep medicine?	Yes	9
	No	91
How many years of experience do you have since you have specialized?	1 to 5 years	40
	5 to 10 years	21
	More than 10 years	39
How long have you been treating OSA patients?	1 to 5 years	62
	5 to 10 years	14
	More than 10 years	24
How often do you come across OSA patients?	Once/week	21
	2 times/week	14
	Once/2 weeks	12
	Once/month	53

OSA = Obstructive sleep apnea.

**Table 2:** Participant’s responses to the knowledge and attitude questions (*n* = 100).

Variables		*n*
Have you come across the topic (Treatment of OSA using OAs)?	Yes	64
	No	36
How do you rate your knowledge level on treatment of OSA using OAs?	Excellent	4
	Adequate	37
	Very good	16
	Inadequate	43
Do you think dentists have a role in helping patients with OSA?	Yes	86
	No	14
Do you know any qualified dentist in dental sleep medicine (received a Fellowship/Board certificate in DSM)?	Yes	13
	No	87
How often do you refer OSA patients to be treated with OAs?	Every 1–4 weeks	4
	Every 1–3 months	9
	Every 4–6 months	13
	Every 6–12 months	12
	Never	62
In cases with high CPAP pressure, have you ever considered a combination treatment (CPAP + OAs) to reduce the required pressure?	Yes	35
	No	65
Do you think OAs have side effects like changes in occlusion (patient’s bite) or teeth position?	Yes	41
	No	11
	I don’t know	48
Do you follow up with your patients whom you referred for OAs treatment?	Yes	31
	No	69
Do you think there are barriers for you to refer cases for OAs?	Yes	59
	No	41

OSA = Obstructive sleep apnea; OAs = oral appliances; DSM = dental sleep medicine; CPAP = continuous positive airway pressure.

**Table 3:** Linear regression model showing the association between the frequency of referral (or not) for oral appliances and experiential factors.

	Unstandardized Coefficients		Standardized Coefficients	t	*p*
	B	Std. Error	Beta		
(Constant)	0.996	0.572		1.741	0.085
Have you come across the topic (Treatment of OSA using OAs)?	−0.550	0.248	−0.228	−2.215	0.029
Knowledge of OAs	0.486	0.111	0.435	4.379	0.000
Do you know any dentists qualified in OSA	0.877	0.299	0.255	2.936	0.004
Do you think OAs have side effects	0.124	0.121	0.101	1.025	0.308
Do you know the mechanism of action of OAs in managing OSA?	0.312	0.226	0.134	1.378	0.171

Dependent Variable: How often do you refer OSA patients to be treated with oral appliances? OSA = Obstructive sleep apnea; OAs = oral appliances; Std. Error = standard error.

## Data Availability

Data available on request from the authors.
